# Identification of resistance in *Escherichia coli* and *Klebsiella pneumoniae* using excitation-emission matrix fluorescence spectroscopy and multivariate analysis

**DOI:** 10.1038/s41598-020-70033-x

**Published:** 2020-08-03

**Authors:** Fernanda S. L. Costa, Caio C. R. Bezerra, Renato M. Neto, Camilo L. M. Morais, Kássio M. G. Lima

**Affiliations:** 1 Institute of Chemistry, Biological Chemistry and Chemometrics, Federal University of Rio Grande do Norte, Natal, RN 59072-970 Brazil; 2 Laboratory of Mycobateria, Department of Microbiology and Parasitology, Federal University of Rio Grande do Norte, Natal, RN 59072-970 Brazil; 3 Lancashire Teaching Hospitals NHS Trust, Fulwood, Preston, PR2 9HT UK

**Keywords:** Applied microbiology, Infectious-disease diagnostics, Pathogens, Computational models, Data mining, Statistical methods, Cheminformatics

## Abstract

*Klebsiella pneumoniae* and *Escherichia coli* are part of the Enterobacteriaceae family, being common sources of community and hospital infections and having high antimicrobial resistance. This resistance profile has become the main problem of public health infections. Determining whether a bacterium has resistance is critical to the correct treatment of the patient. Currently the method for determination of bacterial resistance used in laboratory routine is the antibiogram, whose time to obtain the results can vary from 1 to 3 days. An alternative method to perform this determination faster is excitation-emission matrix (EEM) fluorescence spectroscopy combined with multivariate classification methods. In this paper, Linear Discriminant Analysis (LDA), Quadratic Discriminant Analysis (QDA) and Support Vector Machines (SVM), coupled with dimensionality reduction and variable selection algorithms: Principal Component Analysis (PCA), Genetic Algorithm (GA), and the Successive Projections Algorithm (SPA) were used. The most satisfactory models achieved sensitivity and specificity rates of 100% for all classes, both for *E. coli* and for *K. pneumoniae*. This finding demonstrates that the proposed methodology has promising potential in routine analyzes, streamlining the results and increasing the chances of treatment efficiency.

## Introduction

The Enterobacteriaceae family is one of the most clinically prominent bacteria groups. One of the main gram-negative pathogen is *Klebsiella pneumoniae* (*K. pneumoniae*), which causes opportunistic infections, such as pneumonia, sepsis and inflammation of the urinary tract^1^. Another gram-negative that compose the entereobacteriaceae family is *Escherichia coli*, which are not typically pathogenic to humans and have the ability to cause several diseases in different sites including gastrointestinal tract, the renal system and the central nervous system^2,3^.

Antibiotic therapy induces the selection of resistant bacteria^4^, which generate environmental and health hazards, and economical risk. Over the last decades, several bacterial strains have become progressively resistant to antimicrobial agents^5^. Bacteria may have natural or acquired resistance. Among the genetic variations that confer resistance in bacteria, the main ones are extended spectrum betalactamases^6^ (ESBL), AmpC production, Carbapenemases production^7^, KPC group and MBL group^5^.

Currently, the standard detection method is culture-based, which is time-consuming and labor intensive, providing a slow detection^8^. Other methods can be used to obtain faster results, such as low cytometry^9^, electrochemical detection^10^, and polymerase chain reaction (PCR)^11^. Near infrared (NIR)^12^, Raman^13^ and Fourier transform infrared (FTIR) spectroscopy^14^ have been also reported for these applications.

To identify if a strain of bacteria have resistance is necessary a test where an isolated culture is submitted at several types of antibiotics. The antibiotic sensitivity behavior of the isolated strains can be determined by disc diffusion method^15^, such as Minimal Inhibitory Concentrations (MIC)^16^ or Minimal Bactericidal Concentrations (MBC)^17^.

Fluorescence spectroscopy has already been used in the detection^18^, structural investigation^19,20^ and in the construction of a DNA biosensor for *E. coli*^21^. Chemometric methods such as Linear Discriminant Analysis (LDA)^22^, Quadratic Discriminant Analysis (QDA)^23^ and Support Vector Machines (SVM)^24^, coupled with the dimensionality reduction algorithm: Principal Component Analysis (PCA)^25,26^,and variable selection algorithms: Genetic Algorithm (GA)^27^ and Successive Projections Algorithm (SPA)^28^, tend to enhance the spectroscopic techniques^29–31^.

This paper brings a new perspective for the differentiation of sensitive and resistant bacteria of *E. coli* and *K. pneumoniae* species using excitation-emission fluorescence spectroscopy allied to multivariate classification methods.

## Results and discussion

**Figure 1:**
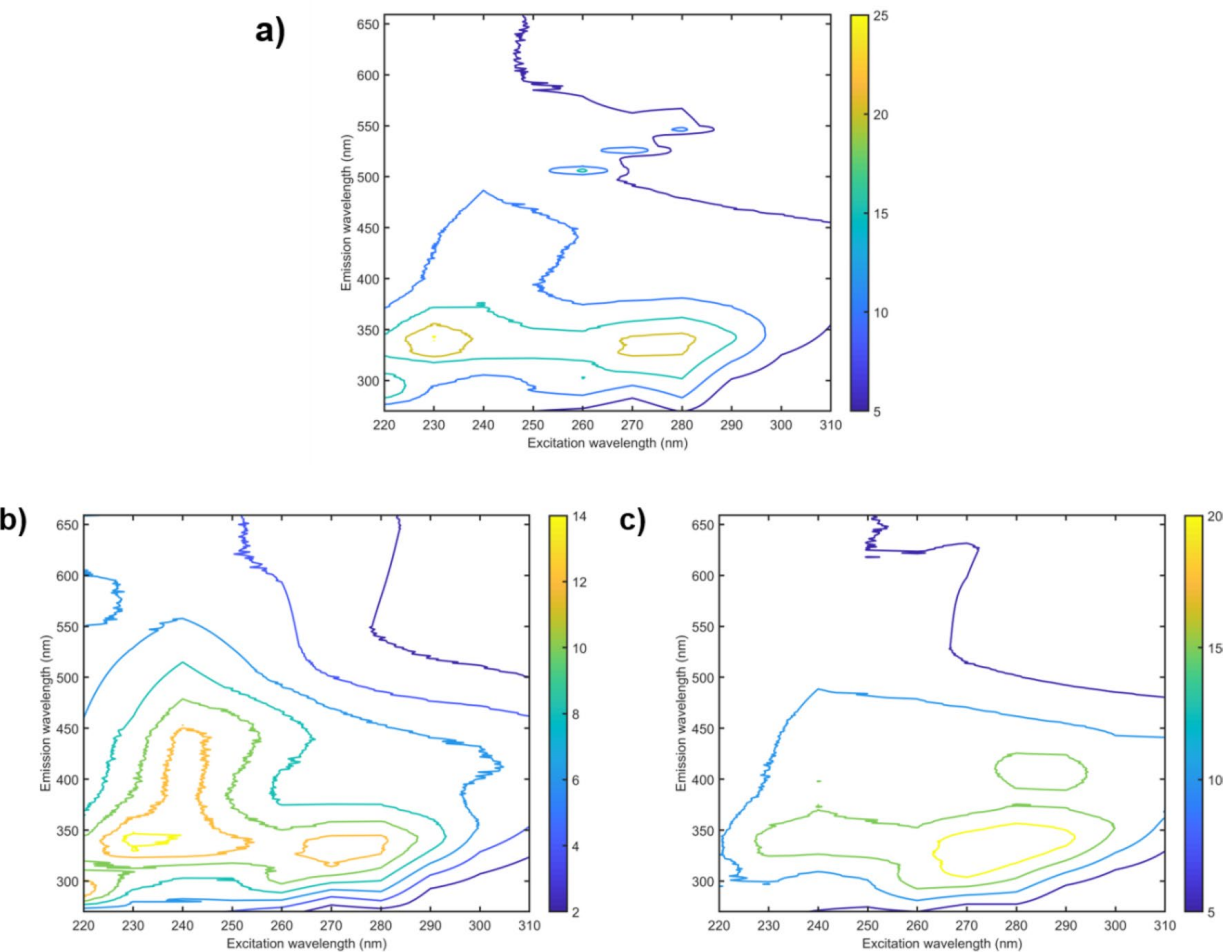
Excitation–emission molecular fluorescence matrix obtained for *Klebsiella pneumoniae*: sensitive (**a**), carbapenems resistant (**b**) and KPC (**c**). The Rayleigh and Raman scatterings were removed from the spectra.

*Klebsiella pneumoniae* samples belonged to three groups, which were named as: Control (ATCC 1706—sensitive samples), resistant 1 (CCBH 6633—samples that show resistance to carbapenems) and resistant 2 (CCBH 4955 KPC—samples resistant to carbapenems, cephalosporins, penicillin). Figure 1 presents the mean excitation-emission fluorescence matrix (EEM) of *Klebsiella pneumoniae:* control (Fig. 1a), carbapenems resistant (Fig. 1b) and KPC (Fig. 1c), after removing Rayleigh and Raman scatterings (the excluded spectral regions were properly corrected by interpolation) and truncation done in the emission matrix.

**Figure 2:**
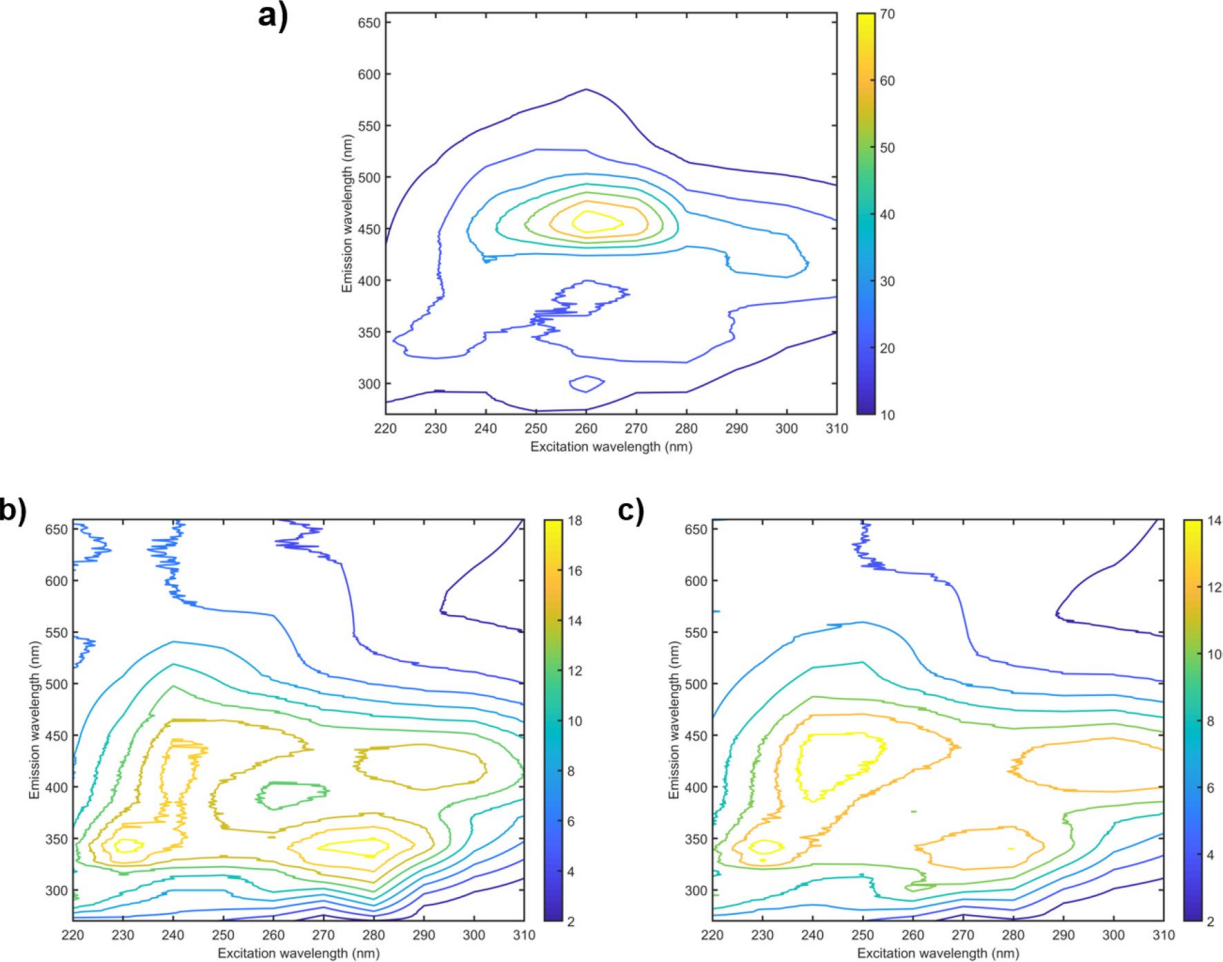
Excitation–emission molecular fluorescence matrix obtained for sensitive *Escherichia coli*: sensitive (**a**), NDM (**b**) and CCBH 7018 (**c**). The Rayleigh and Raman scatterings were removed from the spectra.

The *E. coli* samples were composed of three groups, named control, resistant 1 and resistant 2. The control group was formed by sensitive *E. coli* samples (ATCC 25922). Resistance class 1 was composed of CCBH NDM samples, which have an enzyme called New Delhi metallo betalactamase, which attribute resistance to all beta-lactams, especially carbapenems. The resistant class 2 was formed by *E. coli* CCBH 7018, which shows a type of beta-lactamase that causes hydrolysis of penicillins, monobactams, cephalosporins and cefoxitin. The EEM data obtained for *Escherichia coli*: sensitive (Fig. 2a), NDM (Fig. 2b) and CCBH 7018 (Fig. 2c) are presents in Fig. 2, after spectral pre-processing.

**Figure 3:**
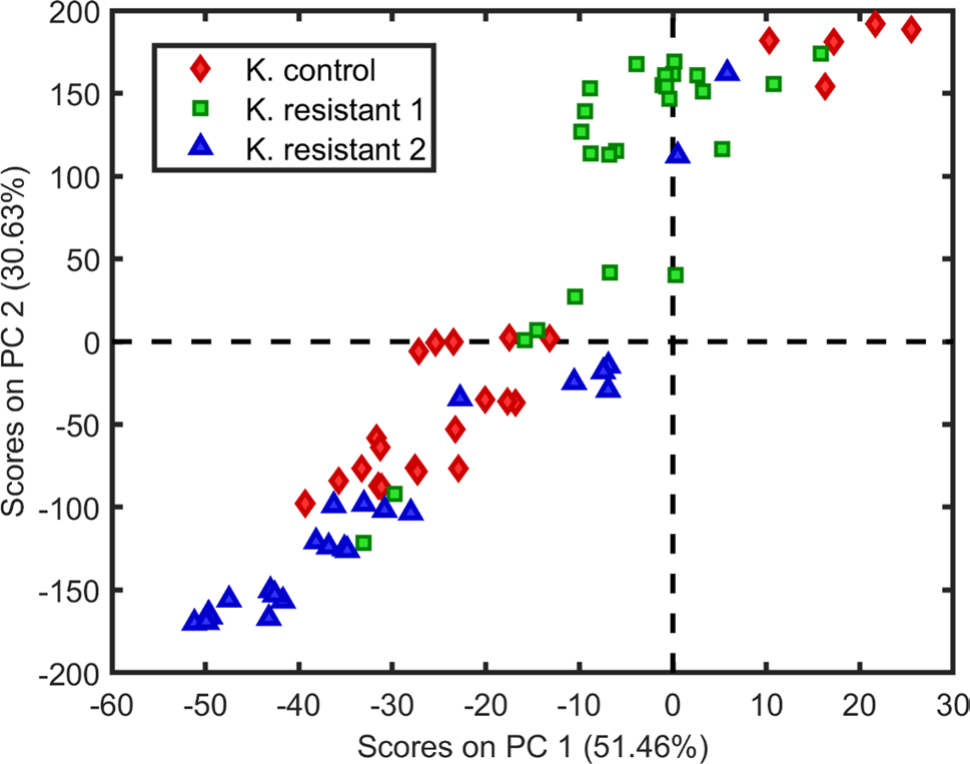
Scores on the first principal component versus the second principal component for classes *Klebsiella pneumoniae:* sensitive (filled rhombus), carbapenems resistant (filled square) and KPC (filled triangle).

As depicted in Fig. 1, it is very difficult to distinguish the classes of sensitive and resistant bacteria only by their spectral profiles due to the great similarity between them. In Fig. 2 there is no such visual similarity, but still, we cannot trace a clear feature that differentiates the classes apart. An exploratory analysis was performed using PCA with the unfolded data after spectral pre-processing. Figure 3 shows the PCA scores for *Klebsiella pneumoniae* data, built with 3 principal components (PCs).

**Figure 4:**
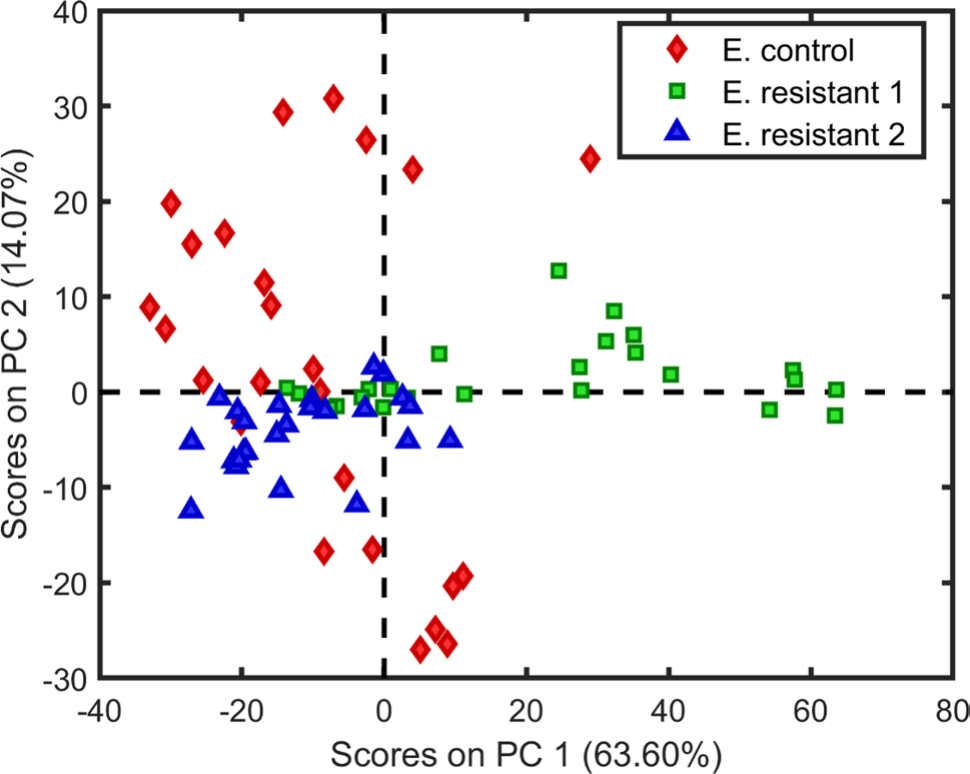
Scores on the first principal component *versus* the second principal component for classes *Escherichia coli*: sensitive (filled rhombus), NDM (filled square) and CCBH 7018 (filled triangle).

It can be observed that in the first component, which explains 51.5% of the explained variance, the control samples do not present separation in relation to the resistant *Klebsiella* samples. The second PC explains 30.6% of the data variance and also fails to distinguish between control and resistant classes. For the *Escherichia coli* spectra, we also constructed a PCA using 4 PCs, where the scores are shown in Fig. 4.

In Fig. 4, it is not possible to identify a separation between the three classes. Projecting the scores for the first PC, which explains 63.6% of the data variance, it is possible to observe a segregation between part of resistance group 1, in relation to the others samples. However, projecting in the second PC, which explains 14.1% of the data variance the data, the three classes cannot be distinguished. PCA results support that it is necessary to use multivariate classification algorithms that maximize the difference between the sensitive and resistant classes. A total of 75 samples were used for building the models, divided into three groups: calibration (45 samples), validation (15 samples) and prediction (15 samples). Table 1 shows the results of classification models built using the EEM fluorescence data for differentiating sensitive *Klebsiella pneumoniae* and resistants *Klebsiella pneumoniae*.

**Table 1:** Results obtained for classification models (2D-LDA, 2D-PCA-LDA, 2D-PCA-QDA, 2D-PCA-SVM, UPCA-QDA/SVM, USPA-QDA/SVM and UGA-QDA/SVM) for sensitive *Klebsiella pneumoniae* and resistant.

Model	Class	Calibration	Prediction
2D-LDA	Control	100.0	100.0
	Resistant 1 + 2	100.0	100.0
2D-PCA-LDA (5)^a^	Control	37.5	62.5
	Resistant 1 + 2	56.5	81.2
2D-PCA-QDA (5)^a^	Control	100.0	93.7
	Resistant 1 + 2	100.0	100.0
2D-PCA-SVM (5)^a^	Control	100.0	100.0
	Resistant 1 + 2	93.8	93.7
2D-LDA	Control	100.0	60.0
	Resistant 1	100.0	100.0
	Resistant 2	100.0	100.0
UPCA-QDA (4)^a^	Control	100.0	100.0
	Resistant 1	100.0	100.0
	Resistant 2	100.0	100.0
USPA-QDA (2)^b^	Control	100.0	100.0
	Resistant 1	93.3	100.0
	Resistant 2	100.0	80.0
UGA-QDA (7)^b^	Control	100.0	100.0
	Resistant 1	100.0	100.0
	Resistant 2	100.0	100.0
UPCA-SVM (4)^a^	Control	100.0	60.0
	Resistant 1	100.0	100.0
	Resistant 2	100.0	100.0
USPA-SVM (2)^b^	Control	73.3	100.0
	Resistant 1	80.0	100.0
	Resistant 2	86.7	80.0
UGA-SVM (12)^b^	Control	100.0	100.0
	Resistant 1	100.0	100.0
	Resistant 2	100.0	100.0

^a^Number of principal components.

^b^Number of selected variables.

Initially, models were constructed comparing the class of *Klebsiella* sensitive and that of resistant. For built this last group, samples of two resistant classes are combined. Among these models, the ones that presented the most satisfactory results were 2D-LDA and 2D-PCA-QDA, which obtained 100.0% calibration accuracy and classification rates above 93% in all classes in the prediction set. Models were constructed using the three classes of samples, applying QDA and SVM, coupled to dimensionality reduction algorithms (PCA, SPA and GA) in the unfolded data. With the exception of the USPA-QDA, UPCA-SVM and USPA-SVM models, all others presented satisfactory results, with 100% accuracy, both in calibration and in prediction, for the three classes.

The same strategy was applied to the *E. coli* samples, the results are shown on Table 2. The first models were created with only two classes: *E. coli* sensitive and the combined resistant samples. The results were satisfactory, mainly for 2D-PCA-LDA and 2D-PCA-QDA, which obtained 100.0% accuracy in both classes, both in calibration and in prediction. The models constructed with the three classes presented satisfactory results in the classification. Unfolded models (UPCA-QDA and UGA-QDA) also resulted in 100.0% accuracy in calibration and prediction of the three classes in this comparison.

**Table 2:** Results obtained for classification models (2D-LDA, 2D-PCA-LDA-2D, 2D-PCA-QDA, 2D-PCA-SVM, UPCA-QDA/SVM, USPA-QDA/SVM and UGA-QDA/SVM) for sensitive *Escherichia coli* and resistant.

Model	Class	Calibration	Prediction
2D-LDA	Control	100.0	87.5
	Resistant 1 + 2	100.0	100.0
2D-PCA-LDA (3)^a^	Control	100.0	100.0
	Resistant 1 + 2	100.0	100.0
2D-PCA-QDA (5)^a^	Control	100.0	100.0
	Resistant 1 + 2	100.0	100.0
2D-PCA-SVM (5)^a^	Control	93.7	100.0
	Resistant 1 + 2	100.0	100.0
2D-LDA	Control	80.0	60.0
	Resistant 1	80.0	80.0
	Resistant 2	100	100.0
UPCA-QDA (4)^a^	Control	100.0	100.0
	Resistant 1	100.0	100.0
	Resistant 2	100.0	100.0
USPA-QDA (2)^b^	Control	100.0	100.0
	Resistant 1	100.0	100.0
	Resistant 2	100.0	80.0
UGA-QDA (7)^b^	Control	100.0	100.0
	Resistant 1	100.0	100.0
	Resistant 2	100.0	100.0
UPCA-SVM (4)^a^	Control	93.3	60.0
	Resistant 1	100.0	100.0
	Resistant 2	100.0	100.0
USPA-SVM (2)^b^	Control	100.0	100.0
	Resistant 1	100.0	100.0
	Resistant 2	100.0	80.0
UGA-SVM (5)^b^	Control	100.0	100.0
	Resistant 1	100.0	100.0
	Resistant 2	100.0	100.0

^a^Number of principal components.

^b^Number of selected variables.

Table 3 presents the validation results of the optimized models (UPCA-QDA, UGA-SVM and 2D-LDA) for each classification category of *Klebsiella pneumoniae*. The models that considered three classes (UPCA-QDA, UGA-SVM) showed promising results, with 100.0% sensitivity and specificity rates. Another notable result is the 2D-LDA model, built with only two classes, achieved similar results, with the same 100.0% sensitivity and specificity rates. The parameters accuracy and F-score were all equal to 100.0%, showing that those models are valid to distinguish between different groups of *Klebsiella pneumoniae* bacteria.

**Table 3:** Quality performance values for the three classification methods (UPCA-QDA, UGA-SVM and 2D-LDA with 2 classes) by molecular fluorescence spectroscopy for each category of *Klebsiella pneumoniae*.

	Stage performance features							
	UPCA-QDA			UGA-SVM			2D-LDA	
	Cont	Res. 1	Res. 2	Cont	Res. 1	Res. 2	Cont	Res. 1 + 2
Accuracy	100.0	100.0	100.0	100.0	100.0	100.0	100.0	100.0
Sensitivity	100.0	100.0	100.0	100.0	100.0	100.0	100.0	100.0
Specificity	100.0	100.0	100.0	100.0	100.0	100.0	100.0	100.0
F-score	100.0	100.0	100.0	100.0	100.0	100.0	100.0	100.0

The validation results of the optimized models UPCA-QDA, UGA-SVM and 2D-PCA-QDA for the *E. coli* are illustrated in Table 4. The sensitivity and specificity rates for these models are 100.0% for all the analyzed classes. The accuracy and F-score values also reinforce the model efficiency.

**Table 4:** Quality performance values of three classification methods (UPCA-QDA, UGA-SVM and 2D-PCA-QDA) by molecular fluorescence spectroscopy for each category of *Escherichia coli*.

	Stage performance features							
	UPCA-QDA			UGA-SVM			2D-PCA-QDA	
	Cont	Res. 1	Res. 2	Cont	Res. 1	Res. 2	Cont	Res. 1 + 2
Accuracy	100.0	100.0	100.0	100.0	100.0	100.0	100.0	100.0
Sensitivity	100.0	100.0	100.0	100.0	100.0	100.0	100.0	100.0
Specificity	100.0	100.0	100.0	100.0	100.0	100.0	100.0	100.0
F-score	100.0	100.0	100.0	100.0	100.0	100.0	100.0	100.0

According to the literature, bacterial resistance is usually associated with the ability of bacteria to modify their cellular structure and induce them to produce substances that neutralize the action of antibacterial agents. Satisfactory results from the models using EEM fluorescence data, for the *E. coli* and *K. pneumoniae* bacteria, demonstrate the sensitivity of the technique in detecting variations in the nuclear content of the cells and in the structure of the membranes itself. As reported by Opačić et al*.*^19^, who used fluorescence spectroscopy on structural investigation of the transmembrane C domain of the mannitol permease from *Escherichia coli*, the results showed that the technique was capable to differentiated the structure of EII^mtl^ from structure of a IIC protein transporting diacetylchitobiose. Additionally, Romantsov et. al.^20^ used dynamic data obtained by fluorescence correlation spectroscopy to extract structural information on isolated nucleoids, besides the evaluation of the characteristic size of the structural units in terms of the DNA length and estimation of their spatial dimensions.

## Methods

### Sample preparation

The samples used were: *E. coli* ATCC 25922—Standard strain, *E. coli* CCBH NDM+ , *E.coli* CCBH 7018, *K. pneumoniae* ATCC 1706, *K. pneumoniae* CCBH 4955, KPC and *K. pneumoniae* CCBH 6633 resistant to Carbapenems. The CCBH strains were obtained from the Laboratory of Hospital Infection (LAPIH—Fiocruz/RJ). The ATCC strains belong to LABMIC/DMP—UFRN. Initially the pure samples were pealed in a BHI broth, then kept in the oven for 24 h at 38 °C, so that the bacteria multiplied. The sample was then pealed on a petri dish containing CLED culture medium, which was also kept in the oven for 24 h. Finally, a bacterial mass corresponding to approximately 2 × 10^6^ colony forming units (CFU) was transferred from culture medium to falcon tube with 2 mL of phosphate buffer solution (1 mol/L), obtaining a concentration of 1 × 10^6^ CFU/mL. To assure this concentration the turbidity was compared with the McFarland standard. The initial solution with the concentration of 1 × 10^6^ CFU/mL was diluted in a phosphate buffer solution (1 mol/L) to obtain the following concentrations, 5 × 10^5^ CFU/mL, 1.3 × 10^5^ CFU/mL, 6.3 × 10^4^ CFU/mL and 3,1 × 10^4^ CFU/mL.

### EEM fluorescence spectroscopy

The excitation/emission fluorescence data were acquired in the wavelength range of 220–310 nm for excitation and 270–900 nm for emission, with steps of 10 and 1 nm for excitation and emission, respectively. A RF-5301 Shimadzu spectrofluorometer with a 0.5 mm quartz cuvette was used. The excitation and emission slits were set at 3 and 5 nm, respectively, the speed scan was set to super mode; the photomultiplier tube was set to the medium level and a cell with a fiber optic reflectance probe was used. A total of 1.5 mL of bacterial solution was added to the fluorescence cuvette for reading. The temperature was maintained at 25 °C throughout the experiments. Five replicates of each concentrations were performed.

## Data analysis

### Chemometrics procedure and software

Spectral pre-processing and multivariate classification models were built using MATLAB R2011a software (The MathWorks, Natick, USA), and the PLS Toolbox 7.9.3 package (Eigenvector Research, Inc., Wenatchee, USA). A spectral range between 220–310 nm for excitation and 270–900 nm for emission was used for model construction, with steps of 10 and 1 nm used for excitation and emission, respectively. This resulted in a data matrix size of 10 × 651 for each sample. The spectral pre-processing was composed by a cut in the region of 270–659 nm in the emission range, and by removing Rayleigh and Raman scatterings using the ‘EEMscat’ algorithm^32^.

The following classification methods were utilized: two-dimensional linear discriminant analysis (2D-LDA)^33^, two-dimensional principal component analysis with linear discriminant analysis (2D-PCA-LDA)^34^, quadratic discriminant analysis (2D-PCA-QDA)^34^, and support vector machines (2D-PCA-SVM)^34^. In addition to these, first-order classification using LDA, QDA and SVM were used in conjunction with the output from the dimensionality reduction algorithms: PCA, GA and SPA.

For the construction of classification models, the samples were divided into calibration (60%), validation (20%) and prediction (20%) sets using the Kennard-Stone (KS) sample selection algorithm^35^. The proposed models were evaluated by calculating some quality parameters such as accuracy, sensitivity, specificity and F-score.

To statistically evaluate the classification models, calculations of sensitivity and specificity were performed using the test samples as important quality measures of model accuracy. Both parameters have a maximum value of 100 and a minimum of 0, and are obtained as follows:

1 $$\mathrm{Sensitivity }\left(\mathrm{\%}\right)=\frac{\mathrm{TP}}{\mathrm{TP}+\mathrm{FN}}\times 100$$

2 $$\mathrm{Specificity }\left(\mathrm{\%}\right)=\frac{\mathrm{TN}}{\mathrm{TN}+\mathrm{FP}}\times 100$$

where FN is defined as a false negative and FP as a false positive; and TP and TN are defined as true positive and true negative, respectively.

Also, the models were evaluated using the area under the curve (AUC) and F-score. The AUC is the area under the receiver operating characteristics conditions (ROC) curve, and the F-score is a measurement of the model accuracy defined by:

3 $$F{\text{-}}score=\frac{2\times SENS\times SPEC}{SENS+SPEC}$$

where SENS stands for sensitivity; and SPEC stands for specificity.

## Conclusion

The present study demonstrates the ability of EEM fluorescence spectroscopy associated with multivariate classification in differentiating classes of susceptible and resistant bacteria of the species *E. coli* and *K. pneumoniae*. The most satisfactory models for the classification of *K. pneumoniae* were UPCA-QDA, UGA-SVM and 2D-LDA, which presented 100% accuracy rates for all classes. For the *E. coli* data, the UPCA-QDA, UGA-SVM and 2D-PCA-QDA models were the best, having 100% predictive performance for the classification of all groups. All these models obtained a sensitivity and specificity rate of 100%. This paper suggest a new alternative in the detection of bacterial resistance, through a methodology that is faster than traditional methods of analysis, simplifying the diagnosis, and increasing the chances of recovery of the patients.

## References

[1] Susanto W, Kong K-H, Hua K-F, Wu S-H, Lam Y (2019). Synthesis of the trisaccharide repeating unit of capsular polysaccharide from *Klebsiella pneumoniae*. Tetrahedron Lett..

[2] Kumar H, Mandal PK (2019). Synthetic routes toward pentasaccharide repeating unit corresponding to the o-antigen of *Escherichia coli* o181. Tetrahedron Lett..

[3] Li X (2019). Disruption of blood-brain barrier by an *Escherichia coli* isolated from canine septicemia and meningoencephali-tis. Comp. Immunol. Microbiol. Infect. Dis..

[4] Mukherjee P (2019). Studies on formulation of a combination heat killed immunogen from diarrheagenic *Escherichia coli* and vibrio cholerae in ritard model. Microbes Infect..

[5] Alharbi NS (2018). Prevalence of *Escherichia coli* strains resistance to antibiotics in wound infections and raw milk. Saudi J. Biol. Sci..

[6] Rodrigues C (2019). Description of *Klebsiella africanensis* sp. nov., *Klebsiella variicola* subsp. tropicalensis subsp. nov. and *Klebsiella variicola* subsp. variicola subsp. nov.. Res. Microbiol..

[7] López-Camacho E (2019). Meropenem heteroresistance in clinical isolates of oxa-48-producing *Klebsiella pneumoniae*. Diagn. Microbiol. Infect. Dis..

[8] Li J, Li B, Liu M (2019). One-step synthesis of mannose-modified polyethyleneimine copolymer particles as fluorescent probes for the detection of *Escherichia coli*. Sens. Actuators B Chem..

[9] Wang X (2014). A metabolomics-based method for studying the effect of yfcc gene in *Escherichia coli* on metabolism. Anal. Biochem..

[10] Ya-li F (2014). Isolation and characterization of an electrochemically active and cyanide-degrading bacterium isolated from a microbial fuel cell. RSC Adv..

[11] Fonseca EL (2017). A one-step multiplex pcr to identify *Klebsiella pneumoniae*, *Klebsiella variicola*, and *Klebsiella quasipneumoniae* in the clinical routine. Diagn. Microbiol. Infect. disease.

[12] Siripatrawan U, Makino Y, Kawagoe Y, Oshita S (2010). Near infrared spectroscopy integrated with chemometrics for rapid detection of *E. coli* atcc 25922 and *E. coli* k12. Sens. Actuators B Chem..

[13] Kusić D, Rösch P, Popp J (2016). Fast label-free detection of legionella spp in biofilms by applying immunomagnetic beads and Raman spectroscopy. Syst. Appl. Microbiol..

[14] Dieckmann R (2016). Rapid characterisation of *Klebsiella oxytoca* isolates from contaminated liquid hand soap using mass spectrometry, FTIR and Raman spectroscopy. Faraday Discuss..

[15] Alam MZ, Aqil F, Ahmad I, Ahmad S (2013). Incidence and transferability of antibiotic resistance in the enteric bacteria isolated from hospital wastewater. Braz. J. Microbiol..

[16] Olorunmola FO, Kolawole DO, Lamikanra A (2013). Antibiotic resistance and virulence properties in *Escherichia coli* strains from cases of urinary tract infections. Afr. J. Infect. Dis..

[17] Levison ME, Levison JH (2009). Pharmacokinetics and pharmacodynamics of antibacterial agents. Infect. Dis. Clin..

[18] Siripatrawan U, Makino Y, Kawagoe Y, Oshita S (2011). Rapid detection of *Escherichia coli* contamination in packaged fresh spinach using hyperspectral imaging. Talanta.

[19] Opačić M, Hesp BH, Fusetti F, Dijkstra BW, Broos J (2012). Structural investigation of the transmembrane c domain of the mannitol permease from *Escherichia coli* using 5-ftrp fluorescence spectroscopy. Biochimica et Biophys Acta Biomembranes.

[20] Romantsov T, Fishov I, Krichevsky O (2007). Internal structure and dynamics of isolated *Escherichia coli* nucleoids assessed by fluorescence correlation spectroscopy. Biophys. J..

[21] Sun J (2015). DNA biosensor-based on fluorescence detection of *E. coli* o157: H7 by au@ ag nanorods. Biosens. Bioelectron..

[22] Liu J, Yu G, Liu Y (2019). Graph-based sparse linear discriminant analysis for high-dimensional classification. J. Multivar. Anal..

[23] Gaynanova I, Wang T (2019). Sparse quadratic classification rules via linear dimension reduction. J. Multivar. Anal..

[24] Tang L, Tian Y, Pardalos PM (2019). A novel perspective on multiclass classification: Regular simplex support vector machine. Inf. Sci..

[25] Tran NM, Burdejová P, Ospienko M, Härdle WK (2019). Principal component analysis in an asymmetric norm. J. Multivar. Anal..

[26] Feng Y, Zhao T, Wang M, Owen D (2018). Characterising particle packings by principal component analysis. Comput. Methods Appl. Mech. Eng..

[27] Islam ML, Shatabda S, Rashid MA, Khan MG, Rahman MS (2019). Protein structure prediction from inaccurate and sparse NMR data using an enhanced genetic algorithm. Comput. Biol. Chem..

[28] Milanez KDTM, Nóbrega TCA, Nascimento DS, Galvão RKH, Pontes MJC (2017). Selection of robust variables for transfer of classification models employing the successive projections algorithm. Anal. Chim. Acta.

[29] Morais CL, Lima KM, Martin FL (2019). Uncertainty estimation and misclassification probability for classification models based on discriminant analysis and support vector machines. Anal. Chim. Acta.

[30] Costa FS (2016). Attenuated total reflection fourier transform-infrared (ATR-FTIR) spectroscopy as a new technology for discrimination between *Cryptococcus neoformans* and *Cryptococcus gattii*. Anal. Methods.

[31] Silva HF (2018). On the synergy between silver nanoparticles and doxycycline towards the inhibition of *Staphylococcus aureus* growth. RSC Adv..

[32] Bahram M, Bro R, Stedmon C, Afkhami A (2006). Handling of Rayleigh and Raman scatter for PARAFAC modeling of fluorescence data using interpolation. Am. J. Chemom. Soc..

[33] da Silva AC (2016). Two-dimensional linear discriminant analysis for classification of three-way chemical data. Anal. Chim. Acta.

[34] Morais CL, Lima KM (2017). Comparing unfolded and two-dimensional discriminant analysis and support vector machines for classification of EEM data. Chemom. Intell. Lab. Syst..

[35] Kennard RW, Stone LA (1969). Computer aided design of experiments. Technometrics.

